# Supplementary figures and images for: Treatment, recurrence rates and follow-up of Tenosynovial Giant Cell Tumor (TGCT) of the foot and ankle—A systematic review and meta-analysis
Source: PLoS One. 2021 Dec 2;16(12):e0260795. doi: 10.1371/journal.pone.0260795 (PMC8638888; doi:10.1371/journal.pone.0260795)

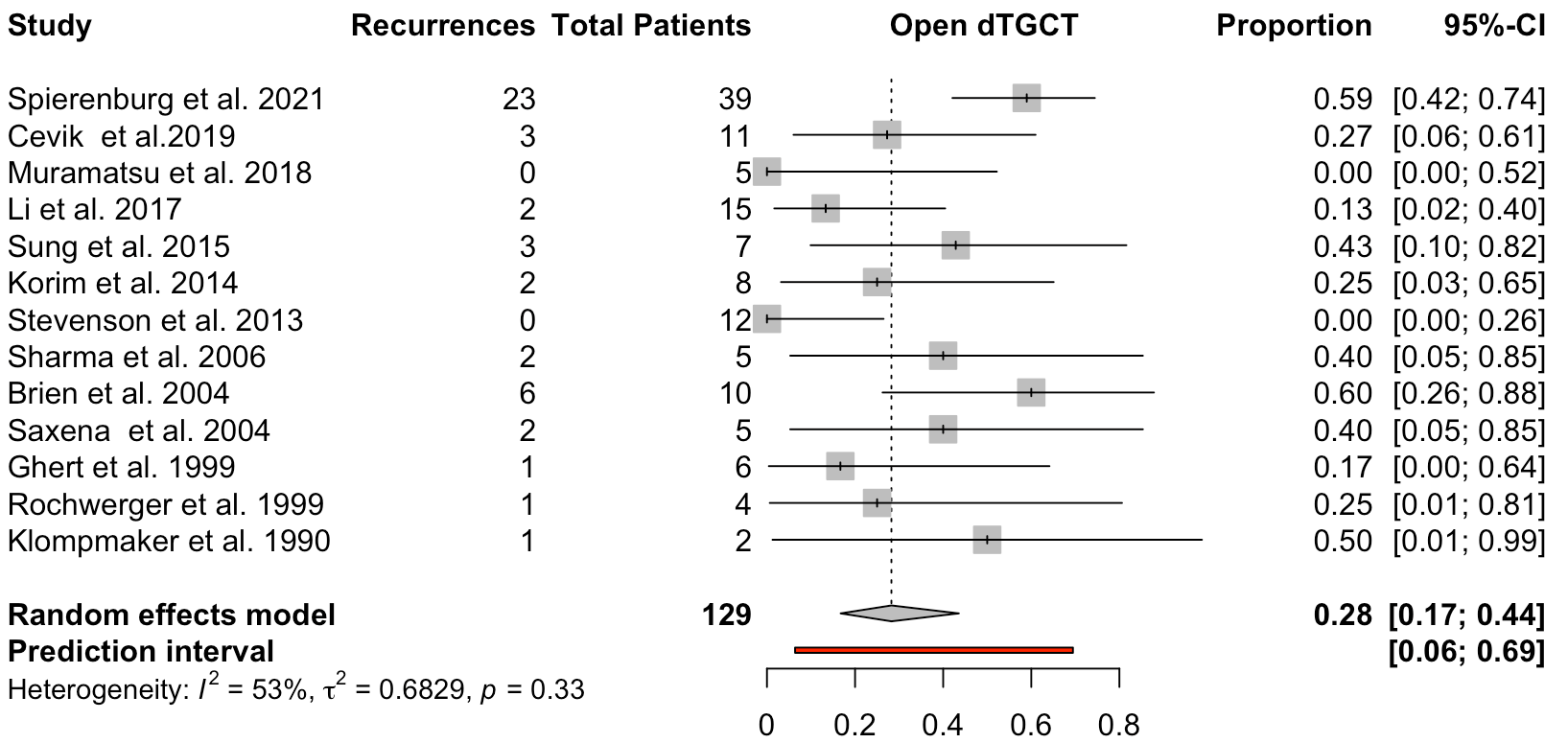

Supplement: S1 Fig — (TIF) [file pone.0260795.s002.tif]

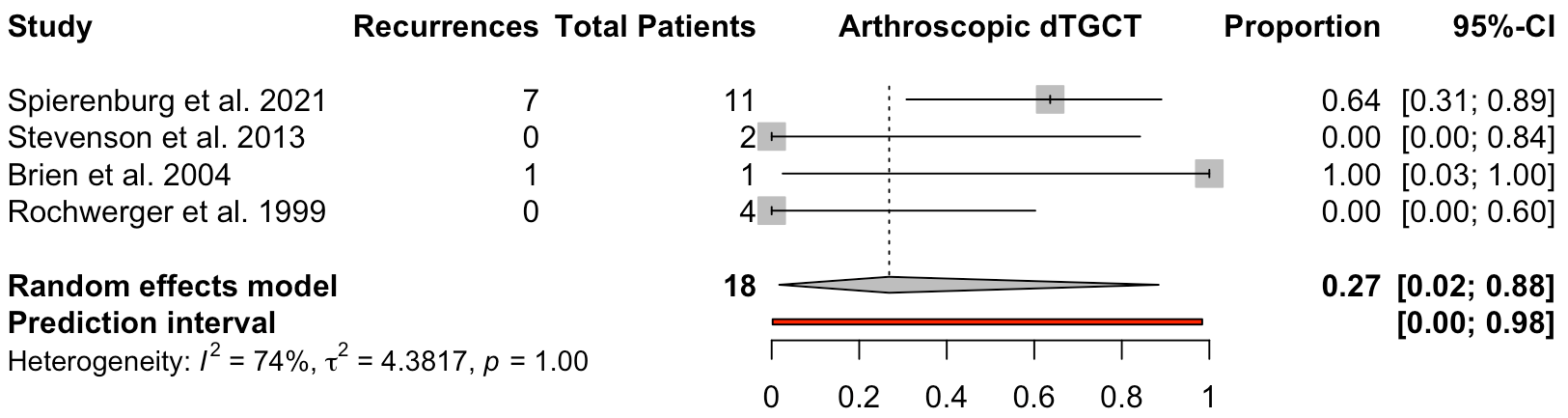

Supplement: S2 Fig — (TIF) [file pone.0260795.s003.tif]

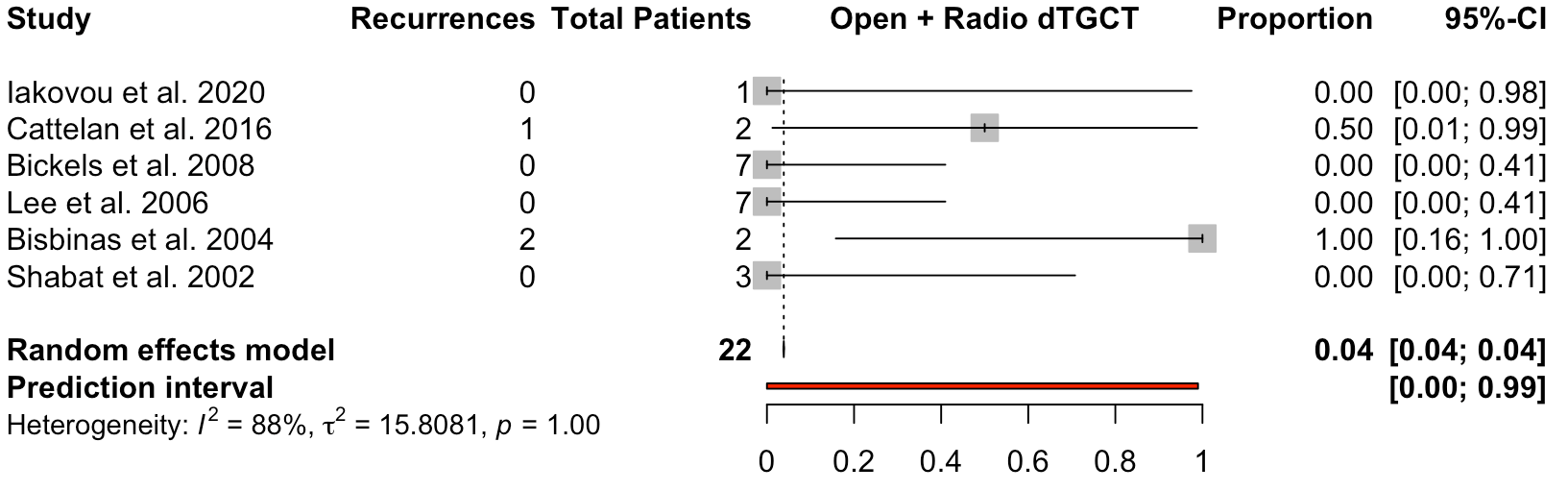

Supplement: S3 Fig — (TIF) [file pone.0260795.s004.tif]

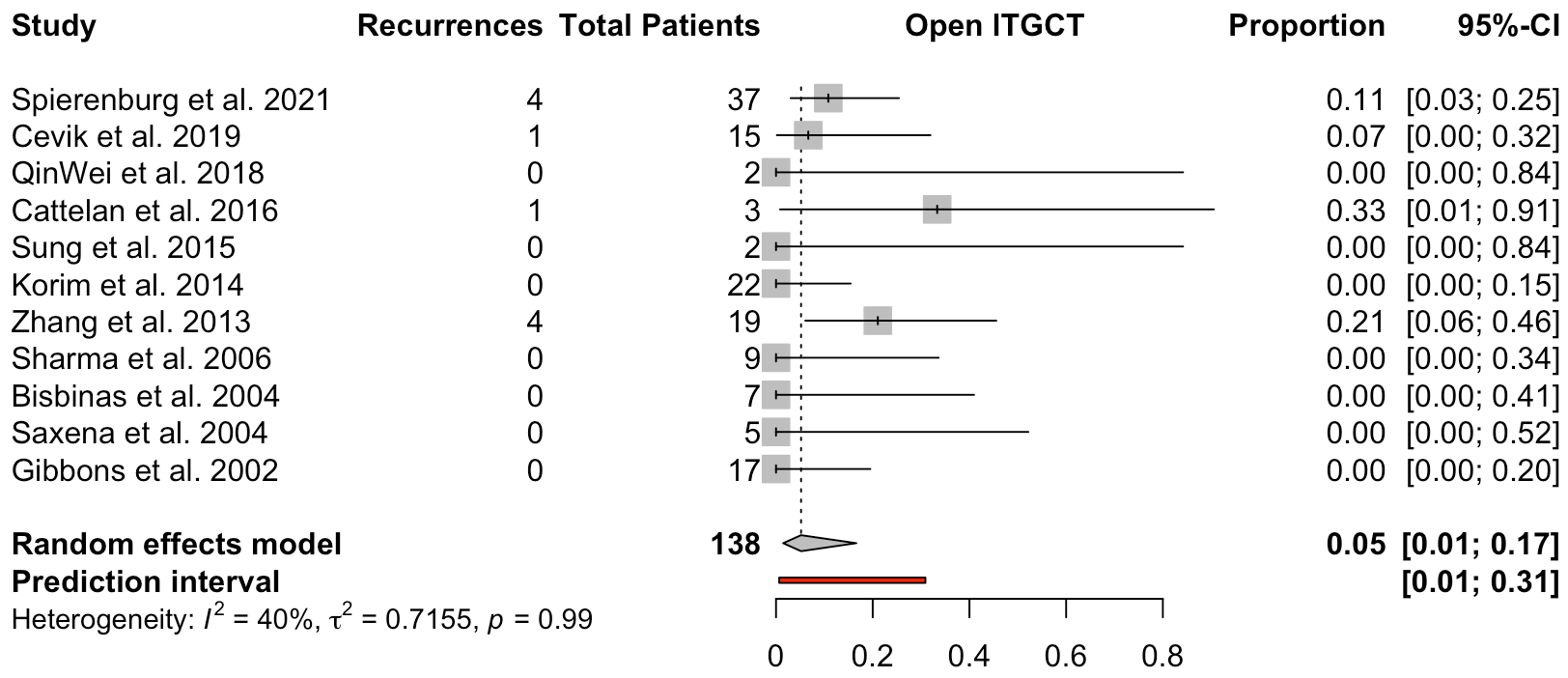

Supplement: S4 Fig — (TIF) [file pone.0260795.s005.tif]

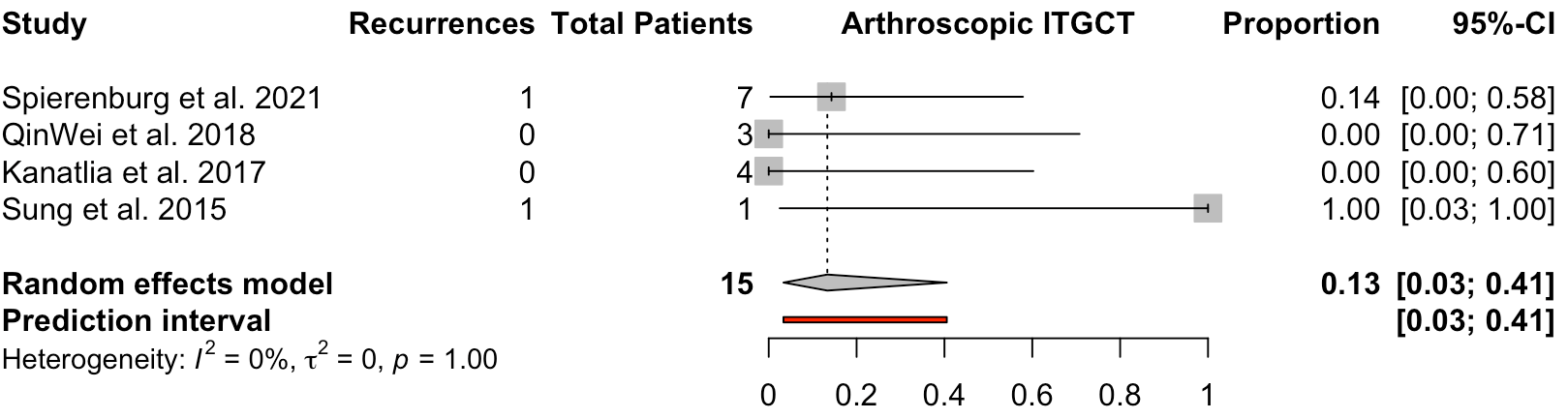

Supplement: S5 Fig — (TIF) [file pone.0260795.s006.tif]
