# Supplementary material for: Treatment, recurrence rates and follow-up of Tenosynovial Giant Cell Tumor (TGCT) of the foot and ankle—A systematic review and meta-analysis
Source: PLoS One. 2021 Dec 2;16(12):e0260795. doi: 10.1371/journal.pone.0260795 (PMC8638888; doi:10.1371/journal.pone.0260795)
Supplement: Attachment — Submitted filename: Point-by-Point-Reponse-Review.docx [file pone.0260795.s007.docx]

*5. Review Comments to the Author
Please use the space provided to explain your answers to the questions above. You may also include additional comments for the author, including concerns about dual publication, research ethics, or publication ethics. (Please upload your review as an attachment if it exceeds 20,000 characters)

Reviewer #1: This review of TSGCTs of the foot and ankle describes the matter clearly and fluently. However, the search items of ‘pigmented villonodular synovitis’, ‘PVNS’ and ‘tenosynovial giant cell’ was not include "giant cell tumor of tendon sheat". I think the result of that choice affect the evaluated articles. As a result, I did not understand why the works I added below were not included in this review.*

**🡪 We have once again revised and updated our search strategy. "Giant cell tumor of tendon sheat" was added as a term.**

**Gibbons, C. L. M. H., Khwaja, H. A., Cole, A. S., Cooke, P. H., & Athanasou, N. A. (2002). Giant-cell tumour of the tendon sheath in the foot and ankle. The Journal of bone and joint surgery. British volume, 84(7), 1000-1003.
**Zhang, Y., Huang, J., Ma, X., Wang, X., Zhang, C., & Chen, L. (2013). Giant cell tumor of the tendon sheath in the foot and ankle: case series and review of the literature. The Journal of Foot and Ankle Surgery, 52(1), 24-27.
***Çevik, H. B., Kayahan, S., Eceviz, E., & Gümüştaş, S. A. (2020). Tenosynovial giant cell tumor in the foot and ankle. Foot and Ankle Surgery, 26(6), 712-716.*

**🡪 These studies are now all included**

*Apart from this small nuance, your review was quite sufficient and full of information.
Yours sincerely

Reviewer #2: I read this manuscript with great interest, however, I had some concerns about this paper which are stated below:

Major revisions:
Methods:
Firstly, I would be interested in the search strategy since I feel the current search is incomplete and does not include all relevant foot/ankle papers. The authors included only three terms, however, they did not include “Giant Cell Tumour of Tendon Sheath” for example. Additionally, several Foot/Ankle TGCT papers were not included, which should have been, such as:
• Gibbons CL, Khwaja HA, Cole AS, Cooke PH, Athanasou NA. Giant-cell tumour of the tendon sheath in the foot and ankle.
• Chen L. Giant cell tumor of the tendon sheath in the foot and ankle: case series and review of the literature.
• Çevik HB, Kayahan S, Eceviz E, Gümüştaş SA. Tenosynovial giant cell tumor in the foot and ankle.
I would suggest the authors to perform a new, more extensive literature search and to update their search to June 2021 when they perform a new literature search, since recently, the largest foot/ankle series is reported (Spierenburg et al.). This new search will add more studies and increase the number of patients for this meta-analysis considerably.*

**🡪 These studies are included, the search algorithm is extended and Spierenburg et. Al is also included.**

*Secondly, the authors chose to evaluate the quality of the studies by the Coleman Methodology score. It is unclear to me why they chose this score. This score was primarily developed for surgical outcomes after patellar tendinopathy, but this study evaluates surgical outcomes and other treatment modalities. The authors also mentioned in the limitations that this score is becoming less frequently used. Perhaps other risk of bias tools needs to be considered (e.g. ROBINS-I).*

**🡪 We reconsidered the need for the Coleman score and finally decided to delete it from our manuscript.**

*Random effects meta-analysis were used to estimate the pooled rate of recurrences with hetereogeneity expressed as I-squared. This analysis is appropriately chosen, however, prediction intervals are missing. IntHout J, Ioannidis JPA, Rovers MM, et al Plea for routinely presenting prediction intervals in meta-analysis*

**🡪 The prediction intervals were attached in agreement with our consulting statisticians.**

*Results
It is unclear why the authors report complications and wound infections separately.*

**🡪 From now on, these will no longer be listed separately.**

*Despite the high heterogeneity, it is remarkable that in the subgroup of patients treated with open surgery and radiotherapy, a 95% CI of 0.04-0.04 was reported. These results, however, should be interpret with caution since the numbers of included patients in this subgroup are small.
Calculating a mean time to recurrence for lTGCT based on only two patients (3 and 108 months), does not have any clinical value.*

**🡪 This is put into perspective in the limitations and in the text, Time to recurrence has now become statistically more meaningful due to more data.**

*Discussion
Given the high heterogeneity regarding some outcomes (complications, overall recurrence and recurrence in treatment subgroups), the authors should interpret their results with more caution since there is a paucity of comparative studies.*

**🡪 This was implemented again in the Discussion and Limitations.**

*The authors claim that there has been a trend towards arthroscopic resection recently, but this is not supported by any references.*

**🡪 This was corrected - here TGCTs of the knee were referred to - however there is no trend in the foot and ankle region.**

*The study of Bickels et al showed unacceptable complications following intra-articular isotope injections in the foot and ankle and discontinued use despite zero recurrences. It is important to highlight such complications in benign disease.*

**🡪 This has now been stated.**

*Regarding the follow-up regime, two comments need to be made:
• The authors calculated a mean time to recurrence of 38 months in 24 patients. Based on this finding, they concluded that patients need to be followed for 5 years. However, the mean follow-up of all patients was 43 months, which is only 5 months longer than the mean time to recurrence. Patients with no recurrence at last moment of follow-up, are still able to develop a recurrence after that (as reported by Mastboom). Therefore, this calculation will always be an underestimation of the actual time to recurrence and therefore, the duration of 5 years is questionable.*

**🡪 Due to the new data, the proposed follow-up regime has now also been adjusted. The underestimation of the correct recurrence rates and especially time-to-recurrence was again discussed in detail in the limitations.**

*• It is unclear why the authors claim to follow-up L-TGCT after three months and annually up to five years after intervention. L-TGCT is often removed in toto due to its nodular presentation, has low recurrence rates and non-aggressive behaviour. Routine annual follow-up till five years after intervention may lead to overdiagnosis.*

**🡪 The proposed follow-up regime was again adapted and individualized accordingly.**

*Finally, in the limitations, the authors mention factors that could have influenced recurrence rates but which could not be evaluated. However, they did not mention the factor of involvement of different anatomical locations in the foot and ankle. TGCT located in the ankle joint or tarsalia has different outcomes/prognosis than TGCT located in the digits.*

**🡪 This was also described again in the Limitation**

*Minor revisions:
Introduction
P3.L65-66: More recent studies reported the incidence rates of TGCT (Mastboom / Ehrenstein).
P.3L67-68: The authors claim that intra-articular tumors mainly occur in larger joints, however, L-TGCT is more common and occurs most often in digits
P3.L76-80: Provide more background information about different treatment options.
P3.L85-86: I guess the authors tried to say that Foot and Ankle TGCT contributes to only a small fraction of an already rare disease, however this is not clearly described.
Table 1: Stevens needs to be Stevenson*

**🡪 The Minor Revisions have all been corrected in the appropriate places.**
