# Supplementary material for: Treatment, recurrence rates and follow-up of Tenosynovial Giant Cell Tumor (TGCT) of the foot and ankle—A systematic review and meta-analysis
Source: PLoS One. 2021 Dec 2;16(12):e0260795. doi: 10.1371/journal.pone.0260795 (PMC8638888; doi:10.1371/journal.pone.0260795)
Supplement: Attachment — Submitted filename: Response_to_Reviewers.docx [file pone.0260795.s008.docx]

**Editor Comments:**

*1. Thank you for stating the following financial disclosure:*

*"The author(s) received no specific funding for this work.
The article processing charge was funded by the Baden-Wuerttemberg Ministry of Science, Research and Art and the University of Freiburg in the funding programme Open Access Publishing."*

*e) Please provide an amended Funding Statement that declares *all* the funding or sources of support received during this specific study (whether external or internal to your organization) as detailed online in our guide for authors at http://journals.plos.org/plosone/s/submit-now.*

*f) Please state what role the funders took in the study.  If any authors received a salary from any of your funders, please state which authors and which funder. If the funders had no role, please state: "The funders had no role in study design, data collection and analysis, decision to publish, or preparation of the manuscript."*

*Please send your amended statements by return email; we will change the online submission form on your behalf.*

Answer:
The above OA funding support is all funding received for this study. The funders had no role in study design, data collection and analysis, decision to publish, or preparation of the manuscript.

“The article processing charge was funded by the Baden-Wuerttemberg Ministry of Science, Research and Art and the University of Freiburg in the funding programme Open Access Publishing. The funders had no role in study design, data collection and analysis, decision to publish, or preparation of the manuscript."

*2. Please review your reference list to ensure that it is complete and correct. If you have cited papers that have been retracted, please include the rationale for doing so in the manuscript text, or remove these references and replace them with relevant current references. Any changes to the reference list should be mentioned in the rebuttal letter that accompanies your revised manuscript. If you need to cite a retracted article, indicate the article’s retracted status in the References list and also include a citation and full reference for the retraction notice.*

Answer: The reference list is reviewed now.

**Reviewer Comments to the Author:**

*Please use the space provided to explain your answers to the questions above. You may also include additional comments for the author, including concerns about dual publication, research ethics, or publication ethics. (Please upload your review as an attachment if it exceeds 20,000 characters)*

*Reviewer #1: I read the revised manuscript with great interest again.
I believe the authors addressed the feedback they have received before adequately.
With the new search they included more relevant papers.
Also, the claims they made are now better substantiated.

If the authors would add references to study regarding the results in lines 263 - 288, that would improve their manuscript.*

Answer: References are added now.

*Reviewer #2: The Systematic Review and Meta-Analysis is much better than previous version. I saw that you put a lot of effort into revising this systematic review.
Line 95 "... shoulder (1%) [25], The lTGCT ..." The comma should be dot.
Congratulations for this review.*

Answer: The comma is a dot now.

Best,

Markus Siegel
